# The Critical Management of Spinal Cord Injury: A Narrative Review

**DOI:** 10.3390/clinpract15010002

**Published:** 2024-12-26

**Authors:** Emilio Moreno-González, Antonio Ibarra

**Affiliations:** Centro de Investigación de Ciencias de la Salud (CICSA), Facultad de Ciencias de la Salud, Universidad Anáhuac México Campus Norte, Huixquilucan CP 52786, Estado de México, Mexico; emilio.morenogo@anahuac.mx

**Keywords:** spinal cord injury, acute management, neuroprotection

## Abstract

Spinal cord injury (SCI) is defined as physical damage that alters the function and structure of the spinal cord. Traumatic causes, such as vehicle accidents, falls, and violence, account for 90% of SCI cases. Recent evidence suggests that early intensive care unit (ICU) monitoring improves patient prognosis, highlighting the importance of prompt ICU admission and early decompression surgery. This review includes 50 publications selected based on specific criteria to gather evidence on the current management of SCI in acute settings. Pharmacological agents have been studied for their neuroprotective properties, offering hope for improved neurological outcomes. Several clinical trials are evaluating new pharmacological alternatives for SCI. In conclusion, the current management of acute SCI should focus on standardized treatments addressing ventilatory, cardiopulmonary, and hematologic complications, all of which directly impact long-term neurological and functional prognosis. New neuroprotective agents currently in clinical trials show promising results and should be further studied to determine their role in acute SCI management.

## 1. Introduction

Spinal cord injury (SCI) is defined as a lesion that disrupts the normal anatomy and physiology of the spinal cord [1]. The etiology of SCI can be broadly categorized into traumatic and non-traumatic causes. Traumatic injuries result from external forces applied to the body, causing tissue damage, such as motor vehicle crashes, falls, or violence [2]. Non-traumatic causes include inflammation, autoimmunity, ischemia, toxicity, or compression of the spinal cord by tumors, metastases, abscesses, or malformations [3].

Cardiac dysfunction and respiratory insufficiency are common in the first 7 to 14 days post-injury, requiring advanced management with continuous hemodynamic and respiratory monitoring [4]. Recent evidence suggests that early ICU monitoring positively affects neurological outcomes, underscoring the importance of prompt ICU admission [5]. Current critical SCI management includes early neuroprotective therapy to improve long-term neurological outcomes [6].

In North America, the prevalence of SCI is approximately 39–40 cases per million people, equivalent to 17,000 to 18,000 cases per year [3]. The increasing prevalence of SCI necessitates the standardization of treatment protocols in the ICU. This review aims to summarize the most recent and relevant information on the acute management of SCI.

The most recent evidence suggests that early-stage treatment and monitoring of SCI patients directly decrease mortality and improve long-term neurological outcomes. SCI significantly impacts patients’ quality of life, leading to years of disability and substantial societal costs. Therefore, this review aims to consolidate new medical evidence to improve the prognosis for SCI patients.

## 2. Methods

**Figure 1:**
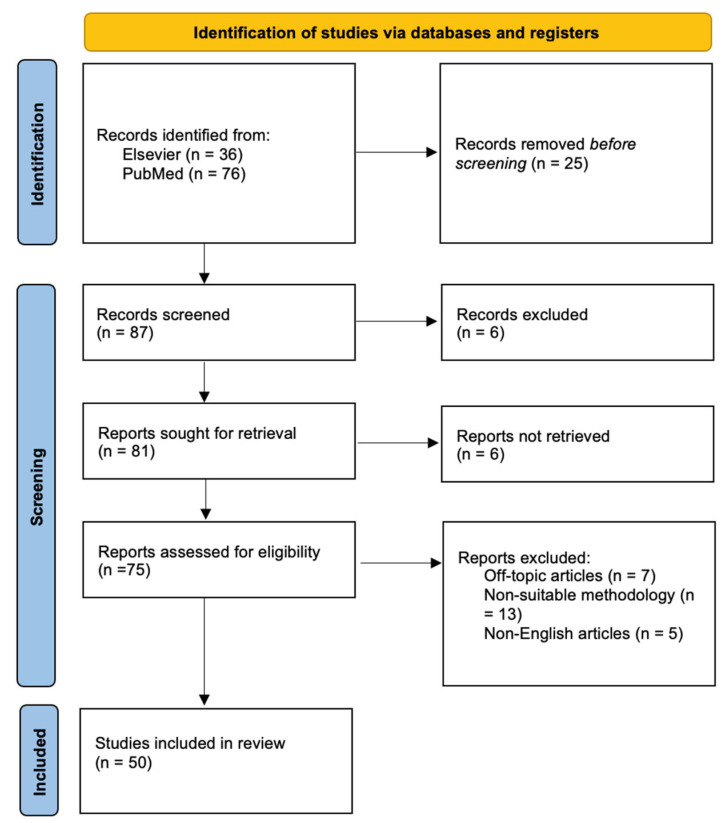
PRISMA flow diagram. A screening of 112 articles was performed, in which 36 Elsevier articles and 76 PubMed articles were collected; only 75 articles were assessed for eligibility and 25 were eliminated.

A literature search was conducted using PubMed (Bethesda, MD, USA) and Elsevier (Maryland Heights, MO, USA) (Scopus) databases from 2019 to 2023, employing keywords such as “Spinal Cord Injury”, “Acute Spinal Cord Injury”, “Critical Care Management”, “Acute Spinal Cord Injury Complications”, “Neurological Emergency”, and “Neuroprotective Agents.” Two authors screened 112 articles: 36 from Elsevier and 76 from PubMed. A total of 75 articles were assessed for eligibility, and 25 were excluded, as shown in the PRISMA diagram below (Figure 1). Both authors reviewed the selected articles to identify key information for this review. No automation tools were used in the process. Only 50 publications were selected with the criteria mentioned previously and that discussed etiology, prevalence, pathophysiology, clinical manifestations, and diagnosis of SCI in general aspects; then, specific articles were selected that discussed hemodynamic, respiratory, and surgical management in the ICU. To gather evidence about new advances the early management of spinal cord injury that had an impact on patients’ mortality and long-term prognosis.

The inclusion criteria were articles published in Neuro-critical, Critical Care, Neurotrauma, Orthopedic, Trauma, Neurosurgery, and Neurology journals within the last five years; written in English; and must include clinical trials, cohort studies, meta-analyses, and animal models. Articles focusing on long-term care, rehabilitation, psychological impact, economic aspects, nursing, or ethical considerations were excluded.

## 3. Results

### 3.1. Epidemiology and Global Statistics

The number of incidences in North America is approximately 39–40 cases per million people, equivalent to 17,000 to 18,000 cases annually [7]. Traumatic causes, such as vehicle accidents, falls, and violence, account for 90% of SCI cases [8]. Two age groups are most affected: young adults (19–30 years, 38.5%) and the elderly (over 70 years, 21.5%) [9]. The Global Burden of Disease Study estimated an incidence number of 0.9 million SCI cases and a prevalence of 20 million cases, leading to 6.2 million years of disability [8,9]. Males are disproportionately affected (80% of cases), with a male-to-female ratio of 4.7:1 [10].

### 3.2. Etiology

As mentioned in Table 1 [11], the most frequent mechanisms of traumatic injury to the spinal cord are traffic accidents such as automobile, bus, train, tractor, or motorcycle crashes, high energy falls (>3 ft), gunshots, physical contact or stabbing, sports-related injury (snow skiing, surfing, football, or wrestling accidents), and medical-related injuries such as adverse effects of medical or surgical procedures. The most common level of injury is the cervical spine (50–60%), followed by the thoracic (30–32%) and lumbo-sacral regions (9%) [10]. Non-traumatic causes include spinal metastases (33.3%), myelitis, spinal multiple sclerosis (22.2%), spinal stenosis (19.4%), ischemia or hematoma (11.1%), and spinal abscesses (5.56%) [3].

### 3.3. Mortality and Prognosis

Despite advances in SCI management, mortality in hospital settings ranges from 4% to 18% [12]. In prehospital settings, 15–20% of patients die before reaching the hospital [10]. Factors increasing mortality risk include higher injury levels, age over 70, concomitant trauma (brain, thorax, abdomen, or extremities), and high-energy mechanisms (e.g., motor vehicle accidents, falls from >3 feet, or ejection from automobiles) [13]. Thromboembolism, multiple organ failure, and respiratory failure are the leading causes of death in the ICU [5].

### 3.4. Physiopathology

Following spinal cord trauma, primary damage is caused by vertebral fractures compressing or dissecting the spinal cord. The first phase occurs within 48 h, during which neurons and oligodendrocytes are damaged, disrupting axonal communication and resulting in partial or complete sensory and motor loss below the injury [14]. The second phase is driven by pro-inflammatory cytokines, including Interleukin (IL)-1α, IL-1β, IL-6, and Tumor Necrosis Factor (TNF), which cause an inflammatory response, ischemia, vascular damage, edema, cellular toxicity, and demyelination, promoting further injury expansion [15]. The subacute phase occurs from 48 h to 7 days post-injury, during which the persistent inflammatory response leads to cell death, cyst formation, and inhibited axonal regrowth [16].

### 3.5. Clinical Presentation

SCI’s clinical presentation depends on the anatomical injury to the spinal cord. Several injury patterns or syndromes are identifiable through distinct manifestations [17]. Central cord syndrome occurs in 15–25% of traumatic SCI cases, with motor and sensory impairment in the upper extremities and bladder dysfunction, typically following hyperextension injuries and common in geriatric patients [18]. Anterior cord syndrome, often associated with flexion injuries, manifests as impaired motor function, pain, and temperature sensation, while proprioception and vibration sense are preserved [19,20]. Posterior cord syndrome presents with preserved motor function but impaired vibration and proprioception senses [20,21]. In hemicord syndrome (Brown-Séquard syndrome), ipsilateral motor function, vibration, and proprioception are impaired, while contralateral pain and temperature sensation are lost [20].

### 3.6. Spinal Shock

Spinal shock refers to the temporary loss of motor and sensory functions below the level of injury due to the depression of spinal cord function. The severity depends on the extent of the spinal cord damage, with the gradual recovery of motor and reflex function occurring over time [21]. The disruption of motor neuron and interneuron communication results in reduced muscle excitability and bladder dysfunction, often requiring catheterization to prevent urinary retention [21].

### 3.7. Neurogenic Shock

Neurogenic shock is a form of distributive shock that results from SCI, leading to a loss of sympathetic tone and unopposed parasympathetic activity, causing hypotension and bradycardia [22]. It is more common in thoracic injuries above T6 and cervical injuries, as the spinal cord’s supraspinal input is essential for maintaining blood pressure [23]. Neurogenic shock causes massive vasodilation, reducing cardiac output and cellular oxygen supply. Hemodynamic criteria for diagnosing neurogenic shock include systolic blood pressure (SBP) below 90 mmHg and a heart rate (HR) below 50 bpm [21,22]. Early identification and the proper management of neurogenic shock in the emergency department are crucial for improving patient outcomes.

### 3.8. Diagnosis

SCI patients must undergo prompt evaluation and transport to the emergency department (ER). The initial assessment follows the airway, breathing, and circulation (ABC) protocol, with proper spinal immobilization using a rigid cervical collar and backboard [15]. A neurological examination is conducted to assess motor, sensory, and reflex functions [5]. Imaging studies such as CT scans are highly sensitive to fractures and dislocations, while MRI is superior for evaluating soft tissues, spinal cord transection, and edema [23]. X-rays are also part of standard imaging in trauma cases, although they are less sensitive to minor fractures and soft tissue injuries [24]. The American Spinal Injury Association’s (ASIA) 2019 classification is used to diagnose and grade SCI based on the completeness of injury and zone of partial preservation [25].

### 3.9. Management in the ICU/Emergency Department

#### 3.9.1. Airway Management

As established by the Advanced Trauma Life Support (ATLS) protocols, the primary approach to SCI patients involves airway management [26]. Cervical injuries at or above C5 carry a high risk of airway compromise due to diaphragmatic innervation loss, requiring mechanical ventilation [20]. Urgent intubation is necessary if a patient exhibits respiratory distress, dyspnea, belly breathing, a vital capacity below 10 mL/kg, or a pCO_2_ greater than 20 mmHg [20,21]. For urgent intubations, video laryngoscopy is recommended to minimize cervical manipulation and prevent further injury. In non-urgent cases, fiberoptic intubation is preferred. Premedication should avoid agents that cause hypotension or bradycardia, as SCI patients are vulnerable to hemodynamic changes. Propofol and thiopental can be used in stable patients, and succinylcholine is recommended within the first 48 h post-injury. Atropine should always be available to manage bradycardia or hypotension, as mentioned in Table 2 [25].

#### 3.9.2. Ventilation Management

SCI patients are at increased risk of hypoventilation due to phrenic nerve paralysis, leading to diaphragmatic dysfunction and impaired secretion clearance, which can cause atelectasis and respiratory distress [25]. Hypoxemia exacerbates neurological injuries by worsening bradycardia and increasing nervous tissue damage. Oxygen saturation should be maintained above 92% to prevent these complications [6]. Approximately 75% of cervical SCI patients require intubation. Evidence suggests that early tracheostomy (within four days of intubation) reduces pulmonary complications, ICU stay, and infection risks, as mentioned in Table 3 [27]. Predictive factors for long-term mechanical ventilation include cervical injuries above C5, complete SCI with ASIA A or B scores, age over 60, and PaO_2_/FIO_2_ ratios below 300 after three days of mechanical ventilation [28].

#### 3.9.3. Hemodynamic Management

Volume loss and neurogenic shock are common in SCI patients. Hypotension, resulting from sympathetic innervation loss and vasomotor tone disruption, worsens spinal cord ischemia [6]. Preventing hypotension by maintaining a mean arterial pressure (MAP) above 85 mmHg for the first week post-injury improves neurological outcomes [4,5,29]. First-line therapy includes volume resuscitation with crystalloid solutions administered via peripheral or central access. If volume resuscitation is insufficient, vasopressors should be used to restore vascular tone. Norepinephrine is recommended for cervical and thoracic injuries, while phenylephrine is preferred for lower thoracic injuries [30,31]. Thromboprophylaxis should also be initiated, as venous thromboembolism (VTE) is a leading cause of mortality in SCI patients. Low-molecular-weight heparin (enoxaparin) at 30 mg twice daily, combined with mechanical prophylaxis, should be started within 72 h of injury and continued for three months as even in Table 4 [32].

#### 3.9.4. Steroid Use in SCI

The use of methylprednisolone has been controversial in SCI management. While its anti-inflammatory properties were once believed to improve neuronal survival, recent studies have shown inconsistent benefits in long-term neurological outcomes. A meta-analysis by Liu et al. involving 1863 patients found that high-dose methylprednisolone administration did not significantly improve motor or sensory recovery. Moreover, it was associated with a higher risk of gastrointestinal hemorrhage and respiratory infections [32]. Another meta-analysis by Geisler et al. similarly concluded that methylprednisolone does not provide significant benefits in a SCI prognosis, presented in Table 5 [33].

### 3.10. Surgical Management

Early decompression surgery for SCI has demonstrated improved neurological recovery, better prognosis, reduced intubation time, and shorter ICU stays in clinical trials [34]. Surgery is highly recommended within 24 h post-injury. However, delays can occur due to the need for medical stabilization. Early surgical intervention is believed to mitigate secondary injury mechanisms and enhance neuroprotection, particularly in the penumbra zone surrounding the injury, preventing further ischemia [34,35]. Therefore, early consultation with a neurosurgical team is crucial when an SCI patient arrives at the ER or ICU.

### 3.11. Gastrointestinal Management

Loss of enteric nervous system control in SCI patients can lead to ileus, gastroduodenal stress ulcers, hemorrhage, pancreatitis, and cholecystitis, often worsened by medications like steroids, antibiotics, and opioids [5]. Gastrointestinal complications should be managed early with H_2_ receptor antagonists, proton pump inhibitors, and manual or digital evacuation of stool. Enteral nutrition should begin as soon as possible, with parenteral nutrition given only when necessary and under close monitoring for metabolic complications. Bladder dysfunction should be managed with catheterization to prevent bladder distension injuries [5]. Regular bowel and urine output monitoring is essential to prevent obstructive and infectious complications that can worsen long-term prognosis.

### 3.12. Subacute ICU Complications

SCI induces pathological and mechanical changes that complicate recovery and increase the risk of life-threatening conditions as patients transition to the chronic phase. Common subacute complications include pressure ulcers, dysreflexia, pneumonia, wound infection, thromboembolism, sepsis, and urinary tract infections as shown in Table 6 [36].

After managing immediate cardiovascular complications, a new homeostasis is typically established, marked by restored blood pressure and heart rate. However, secondary complications such as orthostatic hypotension—a drop in systolic blood pressure > 20 mmHg or diastolic pressure > 10 mmHg upon standing—may develop due to reduced sympathetic innervation and impaired vasomotor regulation, which causes pooling of blood in the lower extremities [37]. Orthostatic hypotension can be aggravated by medications such as diuretics, antidepressants, and narcotics.

Autonomic dysreflexia—a hypertensive crisis caused by unopposed sympathetic innervation, typically triggered by bladder or bowel distension—can also occur. Symptoms include acute hypertension, headache, profuse sweating, and blurred vision. If untreated, it can lead to end-organ damage, including myocardial infarction, intracerebral hemorrhage, or pulmonary edema. Initial treatments include applying nitroglycerine 2% paste above the injury site or administering nifedipine (10 mg every 20–30 min, up to 40 mg in 2 h) [38].

Respiratory complications are also common, with diaphragm, intercostal, and accessory neck muscle dysfunction leading to a loss of vital capacity and impaired coughing, promoting pneumonia, atelectasis, and ventilatory failure. Prevention includes mechanical insufflation and exsufflation devices to clear secretions or manual assistance with coughing in supine or Trendelenburg position [39]. If pneumonia is suspected, patients should receive 7–8 days of antibiotics targeting *Pseudomonas*, Gram-negative bacteria, and *S. aureus* [40].

Pressure ulcers are a common issue due to immobility, most frequently affecting the sacrum, heels, ischium, and occiput. Prevention includes repositioning patients every 2–4 h and using specialized mattresses such as air, gel, or water mattresses. If ulcers develop, treatment should include debridement and topical antibiotics [41].

Urinary tract infections (UTIs) are also common due to urinary retention and catheterization. Antibiotic treatment should be administered when UTIs are present, and bladder drainage should be optimized to prevent infections. Avoidance of long-term catheter use is recommended. Urosepsis, which can develop from UTIs, carries a 15% mortality risk in SCI patients [42].

### 3.13. Future Treatment in the Management of SCI

#### Clinical Trials

Numerous pharmacological agents are being studied for their neuroprotective potential in SCI, with several ongoing clinical trials assessing their efficacy.

- Minocycline, a second-generation synthetic tetracycline, crosses the blood–brain barrier and exerts anti-inflammatory effects by modulating PI3K/Akt, p38 MAPK, inhibiting matrix metalloproteinases, and reducing Ca^2+^ influx. This helps prevent glutamate excitotoxicity. A phase III clinical trial at Rick Hansen Institute (NCT01828203) is evaluating minocycline’s effect on neurological prognosis in traumatic SCI, with results expected to show improvements in motor and neurological scores over placebo [43,44].
- Riluzole, a drug previously used in amyotrophic lateral sclerosis (ALS), Alzheimer’s, and hereditary ataxia, blocks pathological calcium and sodium entry, preventing glutamate-induced excitotoxicity. In animal studies, riluzole has reduced spinal cord ischemia and improved motor outcomes. A phase III trial is currently ongoing to assess its impact on SCI prognosis [45].
- Cethrin, a recombinant C3 transferase variant, inhibits Rho kinase, promoting regeneration and remodeling of cut axons. A phase III trial (NCT02669849) is investigating its long-term neurological outcomes, showing improved motor function in the upper extremities and improved SCI independence measure scores [46].

In addition, cell-based therapies, including Schwann cells, mesenchymal stem cells, and oligodendrocyte progenitor cells, are being investigated for their potential to remyelinate axons and regenerate neural circuits, showing promise in improving motor function in both upper and lower extremities [43,47].

### 3.14. Animal Studies

An experimental study conducted in Kunming investigated the effects of meglumine cyclic adenylate (MCA), a cAMP analog, in promoting motor function and cardiovascular recovery in rats after SCI. MCA improved bradycardia, hypotension, and motor function six weeks post-injury and could be a promising treatment for cardiovascular dysfunction in SCI patients [48].

Another study evaluated the use of rapamycin for treating lung injury after SCI. Rapamycin pretreatment alleviated pulmonary edema, inflammation, and hemorrhage by activating the AMPK-mTORC1 pathway, which promotes autophagy. This study suggests rapamycin could be a potential therapeutic agent for preventing respiratory complications post-SCI [49].

Additionally, research on graphene oxide nanosheet scaffolds showed promise for neuronal regrowth. The scaffolds guided neuronal movement and induced angiogenesis in regenerated tissue. A 10-week follow-up in rats demonstrated that this approach could be an alternative method for promoting axonal regeneration after SCI [50].

### 3.15. Clinical Implications

The collection of up-to-date information on SCI management highlights several clinical implications. Emergency departments should immediately inform ICU staff and consult neurosurgery teams for prompt surgical interventions within the first few hours post-injury. Initial management must address the loss of ventilatory and cardiovascular function through advanced resuscitative measures. New neuroprotective agents, once approved, should be integrated into SCI treatment protocols. Both emergency and ICU teams must establish protocols to administer these medications during acute SCI management, as their timing may be critical to improving outcomes.

The adage “time is spine” should become part of emergency and ICU teams’ mindset when managing SCI, as prompt intervention, both surgical and medical, significantly impacts patients’ long-term prognosis and quality of life.

## 4. Conclusions and Future Directions

Spinal cord injury management, especially the treatment of ventilatory, cardiopulmonary, and hematologic consequences, has a direct impact on the long-term neurological and functional prognosis. The most important advances in the current treatment of spinal cord injury are prompt surgical timing, hemodynamic monitoring, and neuroprotective agents, supported by evidence and clinical trials. Although there is a wide number of clinical trials in several phases testing neuroprotective agents, further research and evidence is needed to identify a specific drug that has an impact on SCI prognosis. Animal model experiments show significant results, and clinical trials should be expected to test the human response to drugs, even though these new strategies have been proven to have a positive outcome, the timing of administration of new drugs should be studied to determine what medications could be used in the acute presentation of spinal cord injury and its acute complications. As many articles mention, “time is spine” must become part of the Emergency Department and Intensive Care Unit mentality when treating spinal cord injury, as prompt surgical intervention and medical management affect a patient’s prognosis and quality of life.

## 5. Limitations

The present narrative review has some methodological limitations, since it only concentrated the search for articles in two databases and excluded articles that were not written in English, that were older than 5 years, and that did not have specific topics. Therefore, due to its methodological nature, this review could exclude articles from other sources, languages, and topics relevant to the subject. Although clinical trials, systematic reviews, meta-analysis, and animal model experiments were chosen, the degree of scientific evidence was variable among the articles chosen, although they met the inclusion criteria, there may be a variation among the degrees of evidence of the information collected. There are also some aspects that were excluded because of the established objectives of the article as they were not relevant to the study.

## Figures and Tables

**Table 1:** Spinal cord injury etiology. Divided into the two main categories with specific disclosure of etiology.

Etiology	Details
Traumatic Causes	Traffic accidents (cars, motorcycles), high-energy falls, violence (gunshots, stabbings), sports injuries (skiing, surfing, football), medical or surgical complications.
Non-Traumatic Causes	Spinal metastases (33.3%), myelitis, spinal multiple sclerosis (22.2%), spinal stenosis (19.4%), spinal ischemia or hematoma (11.1%), meningiomas or neurinomas (8.3%), spinal abscesses (5.56%).

**Table 2:** Airway management in spinal cord injury patients. Indications for intubations and considerations.

Parameter	Details
Indications of urgent intubation	High cervical injuries (above C5), respiratory distress, dyspnea, belly breathing, a <10 mL/kg vital capacity, and pCO_2_ > 20 mmHg
Intubation techniques	Urgent intubation: Video-largyngoscopy to minimize cervical manipulation. Non-urgent intubation: Fiberoptic intubation.
Premedication consideration	Avoid medications that cause hypotension or bradycardia (previously damaged nervous tissue is sensitive to minor changes in perfusion). In case of bradycardia or hypotension, atropine should be used.
Recommended medications for rapid sequence intubations	Hemodynamically stable: Propofol or Thiopental. Neuromuscular blockade: Succinylcholine (within 48 h post-injury).

**Table 3:** Respiratory management of spinal cord injury. Ventilatory strategies, prognosis, and complications.

Parameters	Details
Oxygen therapy goal	Maintain O_2_ saturation > 92% to prevent hypoxemia, bradycardia, and exacerbated neurological injury.
Long-term need for ventilation	Imaging evidence of an injury above C5, complete SCI, ASIA A and B scores, >60 years, and a PaO_2_/FIO_2_ ratio < 300 after 3 days of mechanical ventilation.
Complications due to prolonged ventilation	Increased risk of pneumonia and atelectasis due to impaired cough and secretions

**Table 4:** Hemodynamic management in spinal cord injury. Hemodynamic parameters, approach, and thromboprophylaxis strategies.

Parameter	Details
MAP (Mean Arterial Pressure) Target	>85 mmHg for the first week post-injury to prevent further neurological damage.
Resuscitation Approach	First line: Volume resuscitation with crystalloid solutions. Second line: Vasopressors (norepinephrine recommended for cervical/thoracic injury and phenylephrine for low thoracic injuries).
Thromboprophylaxis	Enoxaparin 30 mg twice daily for 3 months, combined with mechanical prophylaxis (electric calf stimulation).

**Table 5:** Methylprednisolone administration outcomes after SCI. Results of a meta-analysis in which they evaluated the outcome of methylprednisolone in patients with spinal cord injury [31].

Study (Authors)	Sample Size	Use of Methylprednisolone
Liu, Yang, He, Pang, Luo, Liu, Rong.	1863 participants	Not associated with an increase in motor score. RCT: *p* = 0.84; Observational: *p* = 0.44
Geisler, Moghaddamjou, Wilson, Fehlings.	1047 participants	Did not demonstrate an effect in ASIA grade after administration; *p* = 0.331; Fischer’s exact test; two-tailed.

**Table 6:** Complications and management in spinal cord injury patients in the ICU (Intensive Care Unit). Divided by systems, the ICU complications, and their respective treatments.

Complications	Details	Management
Cardiovascular	Orthosthatic hypotension: Drop >20 mmHg systolic or >10 mmHg diastolic when in the upright position. Autonomic Dysreflexia: Hypertension caused by an unopposed sympathetic innervation triggered commonly by bladder or bowel distention.	Orthostatic hypotension: Change position gradually and avoid diuretics, antidepressants, and narcotics. Autonomic Dysreflexia: Nitroglycerine 2% paste or, as an alternative, 10 mg of nifedipine every 20 to 30 min, up to 40 mg in 2 h.
Respiratory	Impaired function of respiratory muscles and protection reflexes (cough and secretion clearance) promotes pneumonia, atelectasis and ventilatory failure in SCI patients.	Prevention: Insufflation and exsufflation devices, mechanically or manually assisted coughing in supine, or Trendelenburg position. Treatment of pneumonia: 7 to 8 days of antibiotics
Urinary Tract	High risk of urinary tract infection (UTI) due to urinary retention and catheterization.	Course of oral antibiotics, proper bladder drainage and avoidance of long-term use of urinary catheter.
Pressure Ulcers	Common due to immobility, often localized in sacrum, heels, ischium, and occiput (all pressure-sensitive areas).	Reposition patients every 2–4 h, use specialized mattresses to prevent ulcers. If present, debride and treat with topical antibiotics.

## Data Availability

All the information used can be found in the citation referenced.
